# Protein functional properties prediction in sparsely-label PPI networks through regularized non-negative matrix factorization

**DOI:** 10.1186/1752-0509-9-S1-S9

**Published:** 2015-01-21

**Authors:** Qingyao Wu, Zhenyu Wang, Chunshan Li, Yunming Ye, Yueping Li, Ning Sun

**Affiliations:** 1 School of Software Engineering, South China University of Technology, Guangzhou, China; 2 School of Computer Science and Technology, Harbin Institute of Technology at Weihai, Weihai, China; 3 School of Computer Science and Technology, Shenzhen Graduate School, Harbin Institute of Technology, Shenzhen, China; 4 School of Computer Engineering, Shenzhen Polytechnic, Shenzhen, China; 5 School of Business and Management, The Hong Kong University of Science and Technology, Hongkong

## Abstract

**Background:**

Predicting functional properties of proteins in protein-protein interaction (PPI) networks presents a challenging problem and has important implication in computational biology. Collective classification (CC) that utilizes both attribute features and relational information to jointly classify related proteins in PPI networks has been shown to be a powerful computational method for this problem setting. Enabling CC usually increases accuracy when given a fully-labeled PPI network with a large amount of labeled data. However, such labels can be difficult to obtain in many real-world PPI networks in which there are usually only a limited number of labeled proteins and there are a large amount of unlabeled proteins. In this case, most of the unlabeled proteins may not connected to the labeled ones, the supervision knowledge cannot be obtained effectively from local network connections. As a consequence, learning a CC model in sparsely-labeled PPI networks can lead to poor performance.

**Results:**

We investigate a latent graph approach for finding an integration latent graph by exploiting various latent linkages and judiciously integrate the investigated linkages to link (separate) the proteins with similar (different) functions. We develop a regularized non-negative matrix factorization (RNMF) algorithm for CC to make protein functional properties prediction by utilizing various data sources that are available in this problem setting, including attribute features, latent graph, and unlabeled data information. In RNMF, a label matrix factorization term and a network regularization term are incorporated into the non-negative matrix factorization (NMF) objective function to seek a matrix factorization that respects the network structure and label information for classification prediction.

**Conclusion:**

Experimental results on KDD Cup tasks predicting the localization and functions of proteins to yeast genes demonstrate the effectiveness of the proposed RNMF method for predicting the protein properties. In the comparison, we find that the performance of the new method is better than those of the other compared CC algorithms especially in paucity of labeled proteins.

## Background

Advances in experimental methods in sequencing technologies results in the rapid growth of genome sequences and gene expression profiles in last decade. A critical problem in making use of these sequenced and associated experimental data is the assignment of functional information. Although the knowledge of protein functions can be acquired by conducting various biochemical experiments, it is both expensive and time-consuming by relying doing experiments alone to identify the functional properties of newly sequenced proteins which can no longer catch up with their rapid growth. Therefore, various computational methods have been developed for automated prediction in the biological literature.

The task of protein functional properties prediction has been explored widely (e.g., see an extensive review on this task for overviews [1]). The conventional prediction methods usually concentrated on protein sequence homology through finding homologies of a protein based on their similarity. Typically, each protein is represented as a feature vector (e.g., textual features from MEDLINE), and the attribute features are taken as input to machine learning algorithms, such as SVM [2], neural networks [3], and random forest [4], to infer annotation rules for predicting the functional properties of unlabeled proteins [5]. However, these kinds of methods do not consider the function diversification when a protein produces interactions with other ones.

Protein-protein interaction (PPI) networks are becoming increasing rich and useful in delineating the biological processes, pathways and complexes that proteins take part in. As a consequence, many works have considered using protein interactions to make prediction. The network-based methods study the task of protein functional properties prediction in the context of PPI networks based on the assumption that the interaction partners of a protein are likely to share similar functions with it. Sharan et al. [6] summarize the methods into two groups: direct annotation schemes, which infer the function of a protein based on its connections in the network [7-9]; and module-assisted schemes which first identify modules of related proteins and then annotate each module based on the known functions of its members [10,11]. However, these types of methods using only interaction partners limit predictions to proteins that have at least one interaction partner with known annotation.

In recent years, there is an increasing concern about using collective classification (CC) that utilizes both attribute features and protein interactions to jointly classifying related proteins in PPI networks [12-14]. CC methods, such as the iterative classification algorithm (ICA), usually explore dependencies between proteins based on the analysis of attributes and functions of neighboring partners. To do so, the attribute features of each protein, together with the additional relational features derived from the linked neighbors are combined for prediction. The additional relational features can potentially increase classification accuracy. But as some of the neighboring proteins' functions may initially unknown, and thus this inferring process may decrease accuracy as well when there are only a limited number of labeled neighboring proteins.

**Figure 1:**
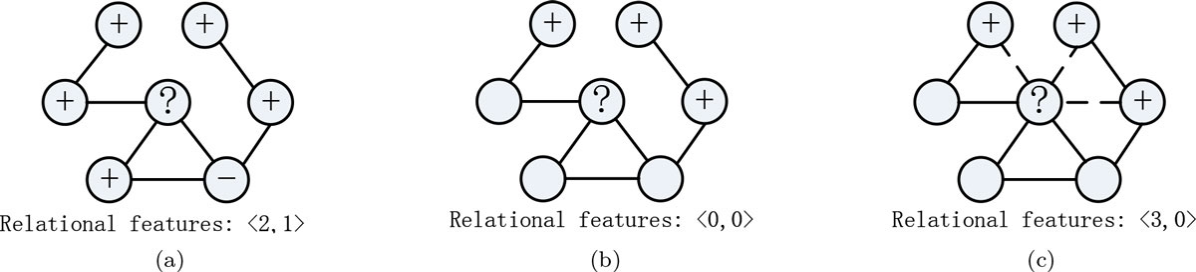
**Label deficiency problem in constructing relational features *x_R _*and latent linkage solution, one approach to generate the relational features is to count the number of neighboring nodes labeled "+" or "-" (for binary problem)**. (a) The unknown protein is linked to 3 labeled protein where 2 are positive and 1 is negative, as such *x_R _*=< 2; 1 >. (b) The labels of neighboring nodes are initially unknown, which makes the compute of relational features very challenging; (c) By adding latent linkages, the unknown protein is connect to the most relevant labeled nodes.

Enabling CC usually improves the performance in protein function annotation, but such a performance improvement usually relies on using a fully-labeled network which contains a sufficient large amount of labeled protein nodes. In this scenario, the labeled neighboring proteins can be used to derive relevant relational features effectively to make prediction (see Figure 1(a)). Indeed, it is difficult and time-consuming to obtain such labels in the protein function prediction field as each protein instance may has multiple functional classes simultaneously. In particular, the number of possible function assignments for a protein is exponential to the number of possible functions in labeling the proteins, which is extremely large even with a small number of possible functional classes. Yet, when one is given only a sparsely-labeled PPI network with limited number of labeled proteins, most of proteins may not directly link to the labeled neighboring proteins. In this situation, relational features based on labels of neighbors is not reliable, and thus learning a CC model with only a few such labels can lead to poor performance (see Figure 1(b)).

To tackle this challenge, we investigate a latent graph approach for finding an integration latent graph by exploiting various latent linkages among protein nodes to link (separate) the proteins with similar (different) functional properties (see Figure 1(c)). Via the latent graph constructed, the supervision knowledge may be able to propagate more effectively from labeled proteins to unlabeled proteins. Then, we develop a regularized non-negative matrix factorization (RNMF) algorithm to make prediction on the latent graph. Conventional non-negative matrix factorization (NMF) method is specifically designed for unsupervised learning and cannot be directly used for network data classification. In RNMF, we extend the NMF objective function by adding a label matrix factorization term and an additional network regularization term to encode the network structure and label information of proteins, and we seek a matrix factorization which gives a new data representation that provides a good approximation of the original data matrix to make prediction for the unlabeled proteins. In this way, the prediction has local smoothness on the network from labeled proteins to unlabeled proteins. As a result, RNMF can have more discriminating power than the ordinary NMF approach which only considers the Euclidean structure of the data.

We study the KDD Cup 2001 tasks of predicting properties (protein localization and their functions) of the protein corresponding to a given yeast gene. Experimental results show that the proposed RNMF algorithm is able to deliver better performance than other compared CC algorithms in paucity of labeled proteins. In summary, the main contributions of this paper are listed as follows:

1 This article studies the protein functional properties prediction problem on sparsely-labeled PPI networks with only a limited number of labeled proteins, which is a very common situation in functional genomics but traditional prediction approaches rarely consider such label deficiency problem.

2 It is the first one to propose a NMF based algorithm that utilizes various additional data sources, including attribute features, latent graph, and unlabeled data, to improve the performance of protein functional properties prediction.

3 The proposed RNMF algorithm extends NMF method by incorporating two additional terms into the model to encode the network structure and labeled information to obtain a local smoothness of predictions on the PPI network. This leads to better prediction performance against the other CC methods especially in paucity of labeled data.

## Methods

### Protein functional properties prediction task

Conventional supervised learning methods assume that the instances to be classified are independent of each other while collective classification (CC) considers to jointly classify interrelated instances in a network by exploiting their interrelations [15]. From this viewpoint, the task of protein functional properties prediction can be cast into the collective classification problem of learning a predictive model from PPI networks. Generally, a PPI network can be represented by a graph where nodes (proteins) interconnected with each other by edges reflecting the interactions between the proteins. Information on each protein node is represented as an attribute feature vector. We are given a set of labeled proteins of known functional classes, and the task is to predict the functions of the remaining nodes of unlabeled proteins. Nevertheless, the functional class membership of one protein may influence the class membership of a related protein.

Formally, the protein functional properties prediction task is described as follows: let **G **= (**V**, **E**, **X**, **Y**) be a protein network dataset. **V **is a set of protein nodes {*v*_1_,...,*v_N_*}. $E=\left[E_{ij}\right]\in {\mathbb{R}}^{N\times N}$ is the weighting matrix whereas *E*_*i*,*j *_indicates the weights on the edge between node *v_i _*and node *v_j _*. $X=\left[x_{\text{1}},\;...,\;x_{N}\right]\in {\mathbb{R}}^{M\times N}$ denotes a data matrix consists of *N *protein attribute feature vectors of dimensionality *M*, where each *x_i _*∈ **X **is an attribute vector for a node *v_i _*∈ **V**. {*c*_1_, *c*_2_, ..., *c_q_*} is the set of *q *possible labels. $Y=\left[Y_{\text{1}},\;...,\;Y_{N}\right]\in {\mathbb{R}}^{q\times N}$ denotes the set of class labels where *Y_i _*is the class labels of protein *v_i_*. Each $Y_{i}={\left[{Y_{i,}}_{\text{1}},\;.\;.\;.\;,Y_{i,q}\right]}^{T}\in {\left\{0,\text{1}\right\}}_{q}$ such that *Y*_*i*,*j *_= 1 means that protein *v_i _*is associated with class *c_j _*and *Y*_*i*,*j *_= 0 otherwise. Assume that we have *n' *labeled proteins ${\left\{\left(x_{i},Y_{i}\right)\right\}}_{i=\text{1}}^{n\prime }$ and *n" *unlabeled data ${\left\{\left(x_{i}\right)\right\}}_{i=n\prime +\text{1}}^{n\prime +n\prime \prime }$ with *N *= *n' *+ *n"*. The task is to predict the functional classes of unlabeled proteins. When there are only a limited number of labeled proteins in the network, i.e. *n' *≪ *n"*, most of the proteins may not connect to labeled ones, which makes the task very challenging. As such, it is natural to consider semi-supervised learning and network exploration techniques to utilize different data sources that are available in this problem setting, including attribute features, protein interactions, and unlabeled data, to improve the prediction performance.

### Nonnegative matrix factorization

Nonnegative Matrix Factorization (NMF) is a matrix factorization technique for discovering low dimensional representations of data [16,17]. In many applications, the input data matrix is of very high dimension, NMF seeks to find two lower dimensional matrices (nonnegative) whose product provides a good approximation to the original data matrix. NMF has received much attention because the learned bases can be interpreted as a natural parts-based representation of data and this interpretation is consistent with the psychological intuition of combining parts to form a whole, like face images and text documents [16,18]. That is, we can explain each data instance by additive linear combination of nonnegative basis vectors because NMF allows only additive combinations. For this reason, NMF has been widely used in various real world applications, such as face recognition [19], document clustering [20] and gene expression analysis [21].

Let $X=\left[x_{\text{1}},\;...,\;x_{N}\right]\in {\mathbb{R}}^{M\times N}$denote the original data matrix with *N *nonnegative column vectors (each is an input instance vector of dimensionality *M*), NMF seeks to find two nonnegative matrices $U=\text{[}u_{ik}\text{]}\in {\mathbb{R}}^{M\times K}$ and $V=\text{[}v_{jk}\text{]}\in {\mathbb{R}}^{N\times K}$ whose product provides a good approximate to the original data matrix **X**, typically *K *≪ *M *and *K *≪ *N*, with the following form

(1) $$X\approx UV^{T}$$

where **U**, **V **≥ 0,**U **is called a basis matrix and **V **is called a coefficient matrix.

The cost function that quantifies the quality of the approximation can be defined in different ways. Here we consider the square of the Euclidean distance of two matrices

(2) $$O=\;||\text{X}-\text{U}{\text{V}}^{T}|{|}^{2}=\underset{i,j}{\sum }{\left(x_{ij}-\overset{K}{\underset{k=1}{\sum }}v_{ik}v_{jk}\right)}^{2}$$

The right hand side of the above objective function is generally positive, vanishing only if the approximation perfectly reconstructs the original data matrix. The above objective function can be minimized by the iterative update algorithm as follows

(3) $$u_{ik}\leftarrow u_{ik}\frac{{\left(XV\right)}_{ik}}{{\left(UV^{T}V\right)}_{ik}}$$

(4) $$v_{jk}\leftarrow v_{jk}\frac{\left(X^{T}U\right)v_{jk}}{{\left(VU^{T}U\right)}_{jk}}$$

The algorithm minimizing the objective function in Eq.(2) using the above multiplicative updates. These updates are guaranteed to decrease the approximation cost at each iteration, and converge to a local minimum of the objective function.

### Latent graphs for protein function prediction

In the protein functional properties prediction task, we are given a PPI network data represented as a graph **G**(**V**, **E**, **X**, **Y**). Our objective is to make prediction for unlabeled proteins on the graph. Recent studies [22,23] have shown that learning performance can be significantly enhanced when the network structure is exploited and the local invariance is considered. The power of these approaches lies in the fact that the exploited network topology generally exhibits the predictable relationships between the input instances and the output class labels.

For protein functional properties prediction, there are two ways of looking at this problem by considering local invariance: i) two neighboring proteins *v_i _*and *v_j _*with large linkage weight *E*_*i*,*j *_are likely to share similar functional classes; ii) if *v_i _*and *v_j _*have small *E*_*i*,*j*_, they tend to have different functional classes. Suppose we have a network structure that can well respect the predictable relationships between the proteins and functional classes, we should be able to have a good performance of predicting the functional properties of unlabeled proteins.

However, the above scheme may not work well on sparsely-labeled PPI networks where there are plenty of links among the proteins but only few of these neighboring proteins are labeled or there are only few links shared between labeled proteins and unlabeled proteins. Recently, researchers [24,25] have considered exploiting various latent linkages among the nodes to find latent graphs with more desirable form of network structures for prediction. Specifically, a weight matrix **E **= [*E_ij_*] is defined for one constructed graph, one latent edge is created for each pair nodes and a weight is assigned on the edge based on the proximity of the nodes. For protein function prediction, we can define the weight matrix **E **for latent graph generation using different data sources that are available in this problem setting. Three of the most commonly used methods are as follows:

*PPI latent graph: *The original PPI network can be considered as a latent graph. We define the weight matrix *E*^(1) ^of the PPI latent graph as follows

$$E_{ij}^{\left(1\right)}=E\left(i,j\right)$$

where *E*(*i*, *j*) = 1 if node *vi *and node *v_j _*are connected in the PPI network, and *E*(*i*, *j*) = 0 otherwise.

*Random walk latent graph: *It is observed that proteins that interact with level-2 neighbors (indirect neighbors in the PPI network) also have a great likelihood of sharing similar characteristics [7]. Thus, we also use the idea of *even-step *random walk with restart (ERWR) [25] to construct the random walk latent graph. Given the weight matrix **E **of the original PPI network, we compute **P **= **EE **and normalize its entries with respect to each column to obtain a normalized transition probability matrix **P**. The ERWR uses a random walker to iteratively visit the neighbourhood nodes with transition probability given in **P**. Also at each step, it has probability *α *(e.g., *α *= 0.1) to return to the start node. We define the weight matrix **E**^(2) ^of the random walk latent graph as follows

$$E_{ij}^{\left(2\right)}=R\left(i,j\right)$$

where $R=\Sigma_{t=\text{1}}^{T}\;\alpha {\left(\text{1}-\alpha \right)}^{t}P^{t}$ is the steady-state probability matrix after *T *steps, and *R*(*i*, *j*) is the (*i*, *j*)th entry in **R**.

*Prediction similarity latent graph: *We consider the values of class labels of the labeled proteins as input features to build a classifier, and give prediction to the remaining proteins. Specifically, we use SVM classifier with probability outputs implemented in the LIBSVM library [26] to compute the classification confidence $Y_{j}^{\prime }$ of a protein *x_i _*to different classes, where $Y_{i}^{\prime }=\left[P\left(c_{\text{1}}\;|\;x_{i}\right),\;\cdot \;\cdot \;\cdot \;,\;P\left(c_{q}\;|\;x_{i}\right)\right]$ where *P*(*c_j_*|*x_i_*) is the probability of the protein *xi *belongs to the class *c_j _*. The weight matrix **E**^(3) ^of latent graph is based on the cosine similarity of prediction confidences of two proteins, and it is defined as follow

$$E_{ij}^{\left(3\right)}=cosin\left(Y_{i}^{\prime },Y_{j}^{\prime }\right)$$

where $cosin\left(Y_{i}^{\prime },Y_{j}^{\prime }\right)=\frac{{Y^{\prime }}_{i}\cdot {Y^{\prime }}_{j}}{\left||{Y^{\prime }}_{i}||\cdot ||{Y^{\prime }}_{j}|\right|}$.

### Latent graphs integration

Our goal is to find a combination of the set of latent graphs {**E**^(1)^, **E**^(2)^, ..., **E**^(*t*)^} to integrate the latent linkages to infer a integration latent graph **Ê **such that the weight on the edge of two protein nodes linked together is large (small) if they have similar (different) functions. Here, *t *is the number of different latent graphs. Formally, we define the integration latent graph **Ê **as follows

(5) $$\begin{array}{c} \hat{E}=\overset{t}{\underset{i=1}{\sum }}w_{i}E^{\left(i\right)}\\ \;\;s.t.\overset{t}{\underset{i=1}{\sum }}w_{i}=1,w_{i}\geq 0 \end{array}$$

where *w_i _*is the combination weight for the *i*th latent graph.

To achieve this, we utilize the latent graph generation algorithm proposed in [24] to learn the weights from the labeled examples, i.e., we only consider the latent linkages among the labeled examples and try to learn the weights from it. Denote a *q*-by-*N *matrix **Y **as the label matrix of all the data, we define another *q*-by-*n' *label matrix **Ῡ **which respects the label information of the labeled examples

(6) $${\bar{\text{Y}}}_{ij}=\left\{\begin{array}{cc} 1, & \text{if the labeled }v_{i}\text{ belongs to }j\text{th class,}\\ 0, & \text{otherwise}. \end{array}\right)$$

where *q *is the number of class labels, *n' *is the number of labeled examples, and *N *is the number of all examples.

Similar, denote a *N*-by-*N *matrix **E**^(*i*) ^as the weight matrix of *i*th latent graph, we define another *n'*-by-*n' *weight matrix **Ē**^(*i*) ^which only respects the linkage weights among the labeled examples of the *i*th latent graph.

We use **Ῡ **and **Ē **that respects the labeled proteins' label information and linkage information to learn the weights. The idea is to ensure that proteins have similar (different) class labels have large (small) linkage weights. To this end, the square of the Euclidean distance between the matrices **Ῡ**^*T*^**Ῡ **and $\overset{q}{\underset{i=1}{\sum }}w_{i}{\bar{E}}^{\left(i\right)}$ is used as the objective function to quantify the quality of the combination weights because the optimal similarity matrix for the labeled examples should be **Ῡ**^*T*^**Ῡ**. Formally, the objective function can be written as

(7) $$\begin{array}{c} \text{min}{∥\overset{t}{\underset{i=1}{\sum }}w_{i}{\bar{E}}^{\left(i\right)}-{\bar{Y}}^{T}\bar{Y}∥}^{2}+\lambda ||\;\text{w}\;|{|}^{2}\\ s.t.\;\;\overset{t}{\underset{i=1}{\sum }}w_{i}=1,w_{i}\geq 0 \end{array}$$

where **Ē **and **Ῡ**^*T*^**Ῡ **are *n'*-by-*n' *matrices, **w **= [*w*_1_, ...,*w_t_*]*^T ^*is the combination weight vector, *λ *is a smoothing parameter (e.g., *λ *= 0.01). The objective is solved to learn the weights **w **for latent graph integration. Then, collective classification methods can be applied on the learnt latent graph to make prediction.

### Regularized NMF with latent graph for protein function prediction

Various methods have been developed for protein functional properties prediction. Previous works have shown that NMF is a general method for robust pattern discovery in complex biological systems [21]. NMF appears to have advantages over other methods such as hierarchical clustering to recover meaningful biological information based on the protein attribute feature matrix. Concretely, NMF aims to find two non-negative matrices whose product provides a good approximation to the original matrix. The nonnegative constraints lead to a parts-based representation. For instance, when applied to face image, NMF yielded a decomposition of faces into parts reminiscent of features such as eyes, nose, etc. The protein patterns in terms of attribute features of the proteins are summarized while applying NMF to the problem of protein function prediction, i.e., NMF is able to provide an interesting decomposition of proteins analogous to facial features in Lee and Seung's work [16] on images.

The main aim of this paper is to study the effectiveness of the NMF and latent graph learning approaches for the problem of protein functional properties prediction. The ordinary NMF method as well as most of its variants cannot be directly applied for network data classification task because the approaches are developed for analysis of unlabeled examples in the context of Euclidean structure of the data. The updates in Eq. (3) and (4) derived from the objective function of NMF in Eq. (2) simply ignore the label information and network structure which play a crucial role for functional genomics problems.

To leverage the power of both NMF and latent graph learning, in this paper, we propose a novel regularized nonnegative matrix factorization (RNMF) algorithm, which seeks a matrix factorization that respects the label information and network structure on the constructed latent graph for protein functional properties prediction. To achieve this, a label matrix factorization term and an additional network regularization term are incorporated into the NMF objective function, and an optimization scheme is developed to solve the objective function of the new NMF method.

Suppose $Y_{i}={\left[{Y_{i}}_{\text{1}},\cdot \cdot \cdot ,Y_{iq}\right]}^{T}\in {\left\{0,\text{1}\right\}}^{q}$ is the label vector of *x_i _*∈ **X**, and $Y=\left[Y_{\text{1}},\;...,\;Y_{N}\right]\in {\mathbb{R}}^{q\times N}$denotes the label matrix encoding the label information of all the data. For labeled data, *Y_ij _*= 1 if *x_i _*is labeled with *c_j _*, and *Y_ij _*= 0 otherwise. For unlabeled data, *Y_ij _*= 0. With the protein attribute feature matrix **X **and label matrix **Y**, the objective function of NMF is extended as follows

(8) $$O=\;||\text{X}-\text{U}{\text{V}}^{T}|{|}^{2}+\;\alpha \;||\;\text{W}⊙\left(\text{Y}-\text{B}{\text{V}}^{T}\right)\;||$$

The above objective function is divided into two terms. The first term is exactly the same as the objective function in Eq. (2), the second term is incorporated into the model to encode the label information, ⊙ is the Hadamard product symbol which is a binary operation that takes two matrices of the same dimensions, and produces another matrix with elements given by [*A *⊙ *B*]*_ij _*= [*A*]*_ij _· *[*B*]*_ij _*, and *α *is a tradeoff parameter to determine the importance of the label matrix term. Here $B\in {\mathbb{R}}^{q\times K}$ is a basis matrix for the second term, and $W\in {\mathbb{R}}^{q\times N}$is a weight matrix such that elements of **W **are with nonzero values if the labels of corresponding proteins are known, otherwise elements of **W **are 0. Specifically, we have

(9) $${\text{W}}_{ij}=\left\{\begin{array}{cc} 0.01, & \text{if}\;Y_{i}\;\text{is}\;\text{known}\;\text{and}\;Y_{ij}=1,\\ 1, & \text{if}\;Y_{i}\;\text{is}\;\text{known}\;\text{and}\;Y_{ij}=0,\\ 0, & \text{if}\;Y_{i}\;\text{is}\;\text{unknown}. \end{array}\right)$$

where *Y_ij _*is either 1 or 0 depending on the class membership of the instance.

In the objective function in Eq. (8), the approximation matrix [**BV***^T^*]*_ij _*(with nonnegative value) does not need to be exactly equal to 1 when *Y_ij _*= 1. On the other hand, we hope [**BV***^T^*]*_ij _*to be close to 0 when *Y_ij _*= 0. Thus, in Eq. (9), the weights with respect to *Y_ij _*= 0 is set to be larger than those of *Y_ij _*= 1 for the labeled data.

By using the new NMF method, the supervised knowledge can be effectively preserved, and we seek a matrix factorization which gives a good approximation for both of the data matrix and label matrix. On the other hand, with the integration latent graph **Ê **constructed, one might further hope that the intrinsic network structure can be considered while applying the NMF method to make prediction. In the following, we incorporate a network regularizer into the NMF objective function to seek a matrix factorization that also respects the intrinsic network structure.

We assume that if the linkage weight *Ê_jl _*of two proteins *x_j _*and *x_l _*on the constructed latent graph **Ê **is large, these two nodes also should be close to each other in terms of the new representations of the matrix factorization. To achieve this, we denote that the new representations of two neighboring nodes *x_j _*and *x_l _*with respect to the new basis matrices are **z***_j _*= [*v*_*j*1_, ..., *v_jK_*]*^T ^*and **z***_l _*= [*v*_*l*1_,...,*v*_*lK*_]^*T*^, respectively. Again, we use the square of the Euclidean distance between these two vectors to measure their distance

$$d\left(z_{j},z_{l}\right)=||\;z_{j}-z_{l}|{|}^{2}$$

With the constructed integration latent graph matrix **Ê **and the distribution distance measure *d*(**z***_j _*, **z***_l_*), we can compute the smoothness of the proteins on the latent graph as follows

(10) $$\begin{array}{c} R=\frac{1}{2}\overset{N}{\underset{j,l=1}{\sum }}||\;z_{j}-z_{l}\;|{|}^{2}\;{Ê}_{jl}\\ =\overset{N}{\underset{j=1}{\sum }}z_{j}^{T}z_{j}D_{jj}-\overset{N}{\underset{j,l=1}{\sum }}z_{j}^{T}z_{l}{Ê}_{jl}\\ =\text{Tr}\left(V^{T}DV\right)-\text{Tr}\left(V^{T}\hat{EV}\right)=\text{Tr}\left(V^{T}LV\right) \end{array}$$

where Tr(·) denotes the trace of a matrix and **D **is a diagonal matrix whose entries are column sum of **Ê**, **D **= ∑*_l _***Ê***_jl_*. **L **= **D **- **Ê **is the graph Laplacian.

Combing this network regularizer  R with the objective function in Eq. (8), we obtain the objective function of RNMF as follows

(11) $$O=||\;X-UV^{T}\;|{|}^{2}+\;\alpha ||\;W⊙\left(Y-BV^{T}\right)\;|{|}^{2}+\beta \text{Tr}\left(V^{T}LV\right)$$

where *β *is the regularization parameter controlling the importance of the network regularization term.

As in the standard NMF, multiplicative updates are derived for **U**, **B **and V for minimizing the objective function. In the following, we introduce an iterative algorithm which can achieve a local minimum for the objective function *O *in Eq. (11). Using the matrix properties Tr(**AB**) = Tr(**BA**) and Tr(**A**) = Tr(**A***^T^*), the objective function can be rewritten as follows

(12) $$\begin{array}{c} O=\text{Tr}\left(\left(\text{X}-\text{U}{\text{V}}^{T}\right){\left(\text{X}-\text{U}{\text{V}}^{T}\right)}^{T}\right)\\ +\;\alpha \text{Tr}\left(\text{W}⊙\left(\left(\text{Y}-\text{B}{\text{V}}^{T}\right){\left(\text{Y}-\text{B}{\text{V}}^{T}\right)}^{T}\right)\right)+\beta \text{Tr}\left({\text{V}}^{T}\text{LV}\right)\\ =\text{Tr}\left(\text{X}{\text{X}}^{T}\right)-2\text{Tr}\left(\text{XV}{\text{U}}^{T}\right)+\text{Tr}\left(\text{U}{\text{V}}^{T}\text{V}{\text{U}}^{T}\right)\\ +\;\alpha \text{Tr}\left(\text{W}⊙\text{Y}{\text{Y}}^{T}\right)-2\alpha \text{Tr}\left(\text{W}⊙\text{YV}{\text{B}}^{T}\right)\\ +\;\alpha \text{Tr}\left(\text{W}⊙\text{B}{\text{V}}^{T}\text{V}{\text{B}}^{T}\right)+\beta \text{Tr}\left({\text{V}}^{T}\text{LV}\right) \end{array}$$

Let *ψ_ik_*, *γ_ik _*and *ϕ_jk _*be the lagrange multiplier for constraint *u_ik _*≥ 0, *b_ik _*≥ 0 and *v_jk _*≥ 0, respectively. We need to minimize  O with respect to **U**, **B **and V subject to the lagrange multiplier constraints. Then we have the Lagrange function  L as follows

(13) $$\begin{array}{c} L=\text{Tr}\left(\text{X}{\text{X}}^{T}\right)-2\text{Tr}\left(\text{XV}{\text{U}}^{T}\right)+\text{Tr}\left(\text{U}{\text{V}}^{T}\text{V}{\text{U}}^{T}\right)\\ +\alpha \text{Tr}\left(\text{W}⊙\text{Y}{\text{Y}}^{T}\right)-2\alpha \text{Tr}\left(\text{W}⊙\text{YV}{\text{B}}^{T}\right)\\ +\alpha \text{Tr}\left(\text{W}⊙\text{B}{\text{V}}^{T}\text{V}{\text{B}}^{T}\right)+\beta \text{Tr}\left({\text{V}}^{T}LV\right)\\ +\text{Tr}\left(\Psi {\text{U}}^{T}\right)+\text{Tr}\left(ϒ{\text{B}}^{T}\right)+\text{Tr}\left(\Phi {\text{V}}^{T}\right) \end{array}$$

where Ψ = [*ψ_ik_*], ϒ = [*γ_ik_*] and Φ = [*ϕ_jk_*].

The partial derivatives of *L *with respect to **U**, **B **and **V **are

(14) $$\frac{\partial L}{\partial \text{U}}=-2\text{XV}+2\text{U}{\text{V}}^{T}\text{V}+\Psi$$

(15) $$\frac{\partial L}{\partial \text{B}}=-2\alpha \left[\text{W}⊙\text{Y}\right]\text{V}+2\alpha \left[\text{W}⊙\text{B}{\text{V}}^{T}\right]\text{V}+ϒ$$

(16) $$\begin{array}{c} \frac{\partial L}{\partial \text{V}}=-2{\text{X}}^{T}\text{U}+2\text{V}{\text{U}}^{T}\text{U}-2\alpha \left[{\text{W}}^{T}⊙{\text{Y}}^{T}\right]\text{B}\\ +2\alpha \left[{\text{W}}^{T}⊙\text{V}{\text{B}}^{T}\right]\text{B}+2\beta \text{LV}+\Phi \end{array}$$

By using the Karush-Kuhn-Tucker conditions *ψ_ik_u_ik _*= 0, *γ_ik_b_ik _*= 0 and *ϕ_jk_v_jk _*= 0, we have

(17) $${\left(\text{U}{\text{V}}^{T}\text{V}\right)}_{ik}u_{ik}-{\left(\text{XV}\right)}_{ik}u_{ik}=0$$

(18) $${\left(\left[\text{W}⊙\text{B}{\text{V}}^{T}\right]\text{V}\right)}_{ik}b_{ik}-{\left(\left[\text{W}⊙\text{Y}\right]\text{V}\right)}_{ik}b_{ik}=0$$

(19) $$\begin{array}{c} {\left(\text{V}{\text{U}}^{T}\text{U}+\alpha \left[{\text{W}}^{T}⊙\text{V}{\text{B}}^{T}\right]\text{B}+\beta \text{DV}\right)}_{jk}v_{jk}\\ -{\left({\text{X}}^{T}\text{U}+\alpha \left[{\text{W}}^{T}⊙{\text{Y}}^{T}\right]\text{B}+\beta \text{EV}\right)}_{jk}v_{jk}=0 \end{array}$$

These equations lead to the following updating rules

(20) $$u_{ik}\leftarrow u_{ik}\frac{{\left(\text{XV}\right)}_{ik}}{{\left(\text{U}{\text{V}}^{T}\text{V}\right)}_{ik}}$$

(21) $$b_{ik}\leftarrow b_{ik}\frac{{\left(\left[\text{W}⊙\text{Y}\right]\text{V}\right)}_{ik}}{{\left(\left[\text{W}⊙\text{B}{\text{V}}^{T}\right]\text{V}\right)}_{ik}}$$

(22) $$v_{jk}\leftarrow v_{jk}\frac{{\left({\text{X}}^{T}\text{U}+\alpha \left[{\text{W}}^{T}⊙{\text{Y}}^{T}\right]\text{B}+\beta \text{EV}\right)}_{ik}}{{\left(\text{V}{\text{U}}^{T}\text{U}+\alpha \left[{\text{W}}^{T}⊙\text{V}{\text{B}}^{T}\right]\text{B}+\beta \text{DV}\right)}_{ik}}$$

when *α *= 0 and *β *= 0 the above updating rules reduce to the updating rules of the original NMF.

**Algorithm 1 **RNMF

**Input: **data matrix **X**, label matrix **Y**, linkage weighting matrix **Ê**, label weighting matrix **W**

**Output: **new label matrix *Y '*

1: Initialize **V **using Eq.(23) and Eq.(25).

2: **repeat**

3:   Update **U **using Eq.(20)

4:   Update **B **using Eq.(21)

5:   Update **V **using Eq.(22)

6:   Reset **V **for labeled data using Eq.(25)

7: **until **stopping criteria is met

8: **for **each unlabeled protein *x_i _***do**

9:   $\hat{k}\leftarrow \text{arg}\underset{k}{\text{max}}\left(v_{ik}\right)$

10:   $Y^{\prime }\left(i,\hat{k}\right)\leftarrow 1$

11: **end for**

The proposed RNMF algorithm is summarized in Algorithm 1. In the algorithm, the first step (line 1) is to initialize the value of **V **for the updates in Eq. (20-22) based on the class priors (using the labeled data). Specifically, for the labeled data we have

(23) $$v_{jk}=\left\{\begin{array}{cc} 1, & \text{if}\;Y_{jk}=1,\\ 0, & \text{if}\;Y_{jk}=0, \end{array}\right)$$

For unlabeled data, the values of *v_jk _*are initialized as

(24) $$v_{jk}=\frac{\sum_{i}n\left(c_{k},x_{i}\right)}{\sum_{k^{\prime }}\sum_{i}n\left(c_{k^{\prime }},x_{i}\right)}$$

where *n*(*c_k_*, *x_i_*) = 1 if *x_i _*is labeled as *c_k _*and 0 otherwise.

The matrices **U**, **B **and **V **are then updated alternately until the objective value of Eq. (11) does not change or the maximum number of iterations is met (line 2-7). In this procedure, the values of *v_jk _*of the labeled data are reset at each iteration to preserve the label information (line 6). In practice, only a small portion of entries of **V **will be reset when we have limited number of labeled data and do not affect the convergence of the algorithm as we see in the experiment section.

By alternatively updating the nonnegative matrices, we obtain a local optimum solution of the coefficient matrix **V**. In the following, we describe how to use **V **for protein function prediction. We specify the column dimension of the new representation $V=\left[v_{jk}\right]\in {\mathbb{R}}^{N\times K}$ of the original data with respect to the new basis as the same as the number of possible class labels *q*, i.e., we set *K *equals to *q*, each dimension of the new representation corresponds to one class label.

For a single label protein *x_j _*function prediction, it is then assigned with the class with the largest *v_jk _*value, i.e.,

(25) $$Y_{jk}=\left\{\begin{array}{cc} 1, & \text{if}\;k=\;\text{arg}\;{\text{max}}_{k^{\prime }}v_{jk^{\prime }},\\ 0, & \text{otherwise}, \end{array}\right)$$

For multi-label protein *x_j _*function prediction, we are primarily interested in learning a model that generate a ranking of possible labels for the given instance such that its correct labels receive higher ranking than the other irrelevant labels. The *v_jk _*value give ranking of labels to indicate the importance of a set of labels associated with the instance. That is, the class label is ordered according to value of *v_jk _*for each instance. A large value of *v_jk _*has a high rank of the corresponding class label. If *v_jk_' > v_jk_"*, the label *k' *is considered to be ranked higher than the label *k"*. The model is then evaluated in terms of its ability to predict a good approximation of ranks for labels associated with the unlabeled instances.

Our proposed RNMF model is different from the other variants of NMF methods. Recently, various researchers have considered manifold learning in matrix factorization. For instance, Cai et al. [22] showed that adding manifold learning in matrix factorization will improve clustering performance substantially. But these NMF methods only deal with unsupervised modeling so far. They cannot directly used for supervised protein functional properties prediction problems where PPI interaction networks are involved. One hopes then to find a matrix factorization which uncovers the network structure and simultaneously respects the label information of the labeled data. In our RNMF model, a label matrix factorization term and a network regularization term are incorporated into the NMF model for this purpose.

## Experiments

In this section, we conduct extensive experiments to compare the performance of our proposed RNMF method with the other compared baselines: SVM, wvRN+RL, ICA, semi-ICA and ICML, and show that the proposed RNMF method is able to achieve better performance against these algorithms.

### Yeast dataset and baselines

We conduct experiments to predict properties of the proteins corresponding to a given yeast gene from KDD Cup 2001 [27] (available at http://www.kdd.org/kdd-cup-2001-molecular-bioactivity-plus-protein-locale-prediction). These properties are (1) the localization of the proteins encoded by the genes (2) one (or several) of categories of protein function(s). A protein can have more than one function, but only one localization. Problem (1) is a binary problem, i.e., proteins are localized (or not localized) to the corresponding organelle. Problem (2) is a multi-label problem with 14 functional classes, and we are primarily interested in learning a ranking of possible functions for the proteins.

The dataset for these two problems includes 1,243 protein instances and 1,806 interactions among the pair of proteins interact with one another. The protein features include the attributes refer to the chromosome on which the genes appears, to whether the gene is essential for survival, observable characteristics of the phenotype, structural category of the protein, the existence of characteristic motifs in the amino acid sequence of the protein, and whether the protein forms larger proteins with others [27,5].

We evaluate the performance of problem (1) by classification accuracy

$$\text{Accuracy}=\frac{\#\text{Unlabe}\text{l}\text{ed data c}\text{l}\text{assified correctly}}{\#\text{Un}\text{l}\text{abe}\text{l}\text{ed data}}$$

and problem (2) by two multi-label learning evaluation metrics *Coverage *and *RankingLoss *[28].

*Coverage *evaluates how far we need, on the average, to go down the list of labels in order to cover all the true labels of an instance:

$$\text{Coverage}(f)=\frac{1}{N}\sum_{i=1}^{N}{}_{c_{k}\in \;Y_{i}}^{\max}\;rank_{s}(x_{i},\;c_{k})-1.$$

where *ranks*(*x_i_*, *c_k_*) denotes the ranks of class label *c_k _*derived from a confidence function *s*(*x_i_*, *c_k_*) which indicates the confidence for the class label *c_k _*to be a proper label of *x_i_*.

*Ranking loss *evaluates the average fraction of label pairs that are reversely ordered for the instance:

$$RankingLoss\left(f\right)=\frac{1}{N}\overset{N}{\underset{i=1}{\sum }}\frac{1}{\left|Y_{i}||{Ȳ}_{i}\right|}\;\cdot \;|R_{i}|,$$

where *R_i _*= {(*c*_1_, *c*_2_)*|h*(*x_i_*, *c*_1_) ≤ *h*(*x_i_*, *c*_2_), (*c*_1_, *c*_2_) ∈ *Y_i _*× *Ῡ_i_*|}, and *Ῡ_i _*denotes the complementary set of *Y_i_*.

1 **SVM **[26]. This baseline is a feature-based method only using the attribute features of the proteins for learning without considering to use any network sources.

2 **wvRN+RL **[29]. This algorithm is a relational-only method only using the PPI network for prediction. wvRN+RL computes a new label distribution for an unlabeled node by averaging the current estimated distributions of its linked neighbors. This process is repeated until reaching the maximum iteration number.

3 **ICA **[15]. This denotes a collective classification algorithm which uses both attribute features and relational features to train a base classifier iteratively for prediction. The relational features are constructed based on the labels of neighbors. We use logistic regression (LR) as base classifier because prior works have found that LR to be superior to other classifiers such as naive bayes and *k*NN, as base classifier for ICA.

4 **semi-ICA **[30]. This method extends ICA to leverage the unlabeled data using semi-supervised learning. There are four semi-ICA variants (KNOWN-EM, ALL-EM, KNOWN-ONEPASS, ALL-ONEPASS) for semi-ICA, we run all four variants and choose the best one as the result of semi-ICA.

5 ICML [31]. This method extends ICA to handle multi-label learning by constructing additional label correlation features to exploit the dependencies among the labels as additional input features to learn base classifier.

It is generally more difficult to determine the classifier parameter values when the number of labeled data available is smaller. Learning from limited number of labeled data is the focus of this study. Thus, we do not tune the algorithm parameters using cross validation. In the experiments, we use default parameter values for the compared methods as recommended by previous works. In particular, we use the LibSVM (available at http://www.csie.ntu.edu.tw/ cjlin/libsvm/) library [26] with linear kernel as base classifier for the SVM algorithm, and set the penalty parameter *C *= 1.0 for the SVM as default. The maximum number of iterations for ICA, semi-ICA are set to 10 as in [30,31]. While the wvRN+RL uses 1000 iterations. The parameters *α *and *β *for our proposed method are set to 10 and 5. The parameter selection will be discussed in the later section.

### Results on protein localization prediction

We first consider problem (1) of KDD Cup 2001, i.e., the protein localization prediction problem. We compare RNMF with the learning algorithms: SVM, wvRN+RN, ICA and semi-ICA. The performance is measured in classification accuracy.

We note that a smaller number of label data is the most interesting case for our algorithm, because it is not reliable for classification prediction due to the inadequacy of supervision knowledge in the labeled dataset. In order to validate the performance of the algorithm in paucity of labeled data, only a small number of proteins are selected as labeled data, which makes the problem very challenging. The remaining are used for testing the quality of the algorithms through the classification accuracy. In the experiments, we use varying number of labeled data ranging from 2% to 5%. For each labeled/unlabeled data split, we execute an algorithm for 10 runs (we have also try 50 runs, the results are similar), and report the performance (mean and standard deviation) over 10 runs for each algorithm. Table 1 shows the experimental results of the algorithms with respect to different ratios of labeled data. One observes that the overall picture taken from the experiments is clearly in favor of our proposed RNMF. The performance of RNMF is consistently better than the other algorithms across different ratios of labeled data. On average, RNMF performs best followed by semi-ICA, these two methods are much better than the SVM method only using attribute features and the wvRN+RL only using relational information.

**Table 1:** Accuracy (mean ± standard deviation) of the compared algorithms against different label ratios on problem (1) of KDD Cup 2001.

label ratio	RNMF	SVM	wvRN+RL	ICA	semiICA
2%	**0.790 ± 0.023**	0.700 ± 0.044	0.633 ± 0.012	0.700 ± 0.058	0.725 ± 0.052
3%	**0.827 ± 0.031**	0.736 ± 0.004	0.624 ± 0.013	0.731 ± 0.063	0.755 ± 0.004
4%	**0.833 ± 0.021**	0.774 ± 0.005	0.650 ± 0.004	0.760 ± 0.052	0.774 ± 0.055
5%	**0.843 ± 0.008**	0.770 ± 0.003	0.675 ± 0.023	0.771 ± 0.058	0.792 ± 0.001
Avg.	**0.823 ± 0.020**	0.745 ± 0.014	0.645 ± 0.013	0.740 ± 0.057	0.762 ± 0.028

We further analyze the performance difference between RNMF and the compared methods and count the results of the win-tie-loss with pairwise t-tests at 0.10 significance level. The label ratios used are 2%, 3%, 4% and 5%. For each label ratio, a win (or loss) is counted when RNMF is significantly better (or worse) than the compared algorithm over 10 runs. Otherwise, a tie is recorded. We find that the win/tie/lose counts with pairwise t-test for RNMF against other algorithms are 4/0/0 over all comparisons. This result reveals that the RNMF method is statistically superior to other methods at 0.10 significance level when there is limited number of labeled data. This is consistent with our earlier assertions that our approach can work well in the paucity of labeled proteins.

**Figure 2:**
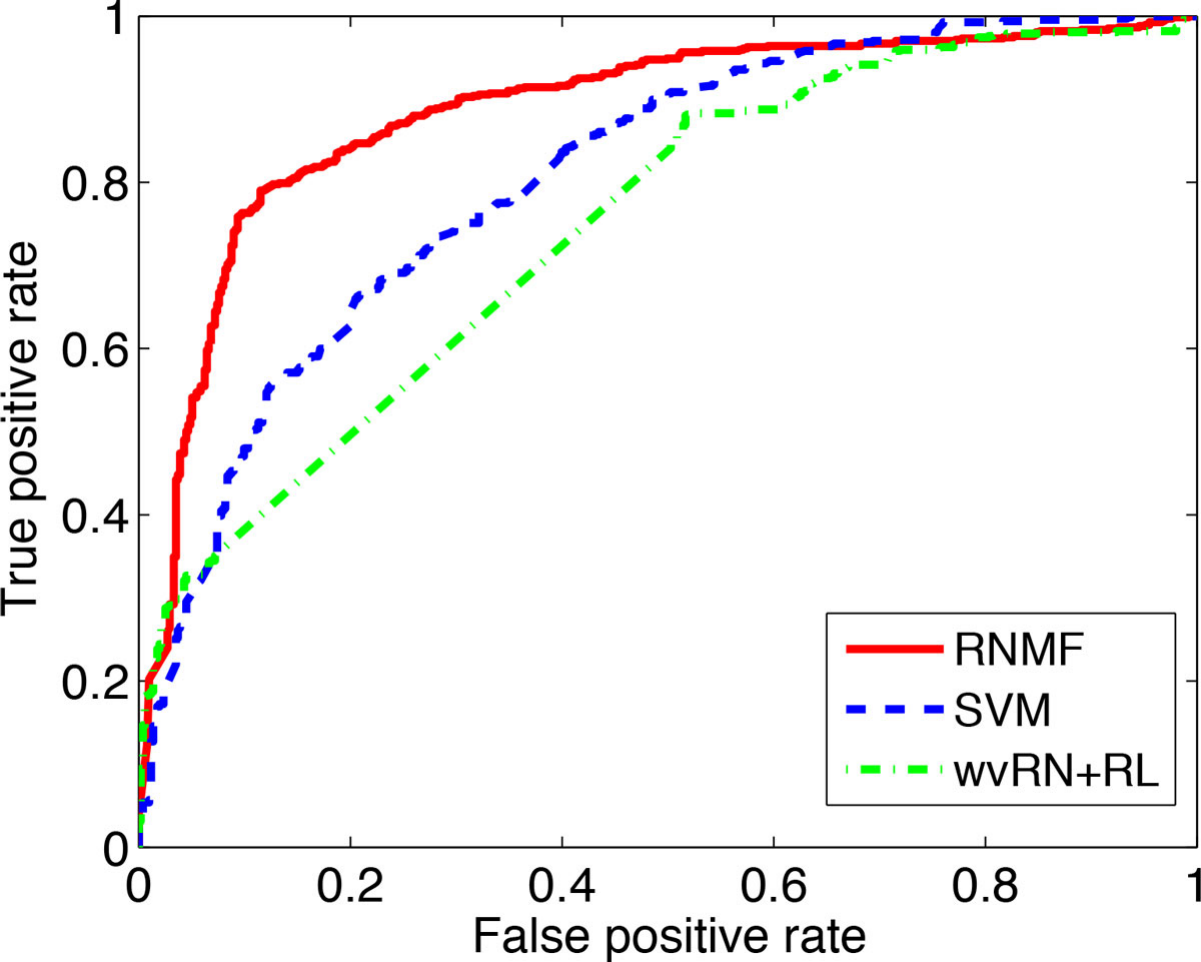
**ROC curves of RNMF and baselines (SVM and wvRN+RL)**.

We also use the receiver operating characteristics (ROC) curve [32] to present results for the protein localization prediction problem with 5% of label ratio. ROC curve reflects the true positive rate of a classifier as a function of its false positive rate. ROC curve is a two-dimensional graph in which false positive (fp) rate is plotted on the *X *axis and true positive (tp) rate is plotted on the *Y *axis. In classification evaluation, the classifier model produces a continuous output (i.e., an estimate of an instance's class membership probability) to which different thresholds are applied to predict class membership. If the classifier output is above the threshold, the classifier predicts the instance as class *c*, else _*c*. In this way, each threshold value produces a different prediction result to compute the results of tp and fp. Each of the thresholds corresponds to a different point in ROC space. The area under the ROC curve (the larger the better) is used to evaluate the strength of a classifier across various thresholds. Figure 2 shows the ROC curves of the RNMF method and the baselines (SVM and wvRN+RL). We see from the figure that the area under the ROC curve of our RNMF (the red curve) is larger than those of the SVM method (the blue curve) and the wvRN+RL method (the green curve), which implies that the RNMF method is able to deliver better performance against the baselines for protein localization prediction.

### Convergence study

**Figure 3:**
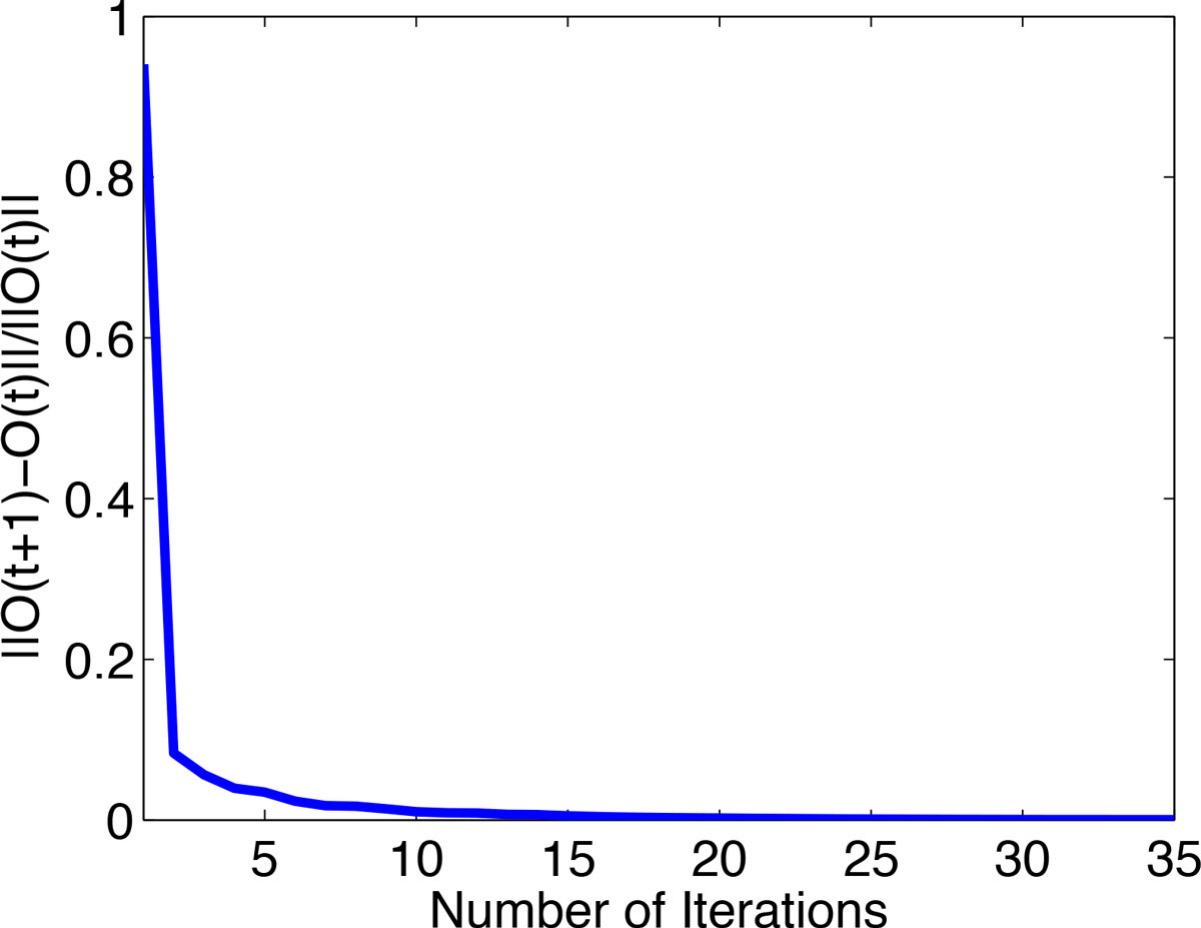
**Convergence curve of RNMF for the problem (1) of KDD Cup 2001 dataset**.

The objective function  O in Eq. (11) is optimized for classification prediction based on the iterative algorithm in Algorithm 1. Here, we investigate how fast the algorithm can converge. Figure 3 shows the convergence curve of the RNMF algorithm on the problem (1) (at 5% label ratio). The *x*-axis is the number of iteration number in the process of optimizing the objective value  O and the *y *axis is the value of successive computed objective value ||O(t+1)-O(t)||/||O(t)||. We observe that the algorithm converge after about 10 iterations.

### Parameter sensitivity

**Figure 4:**
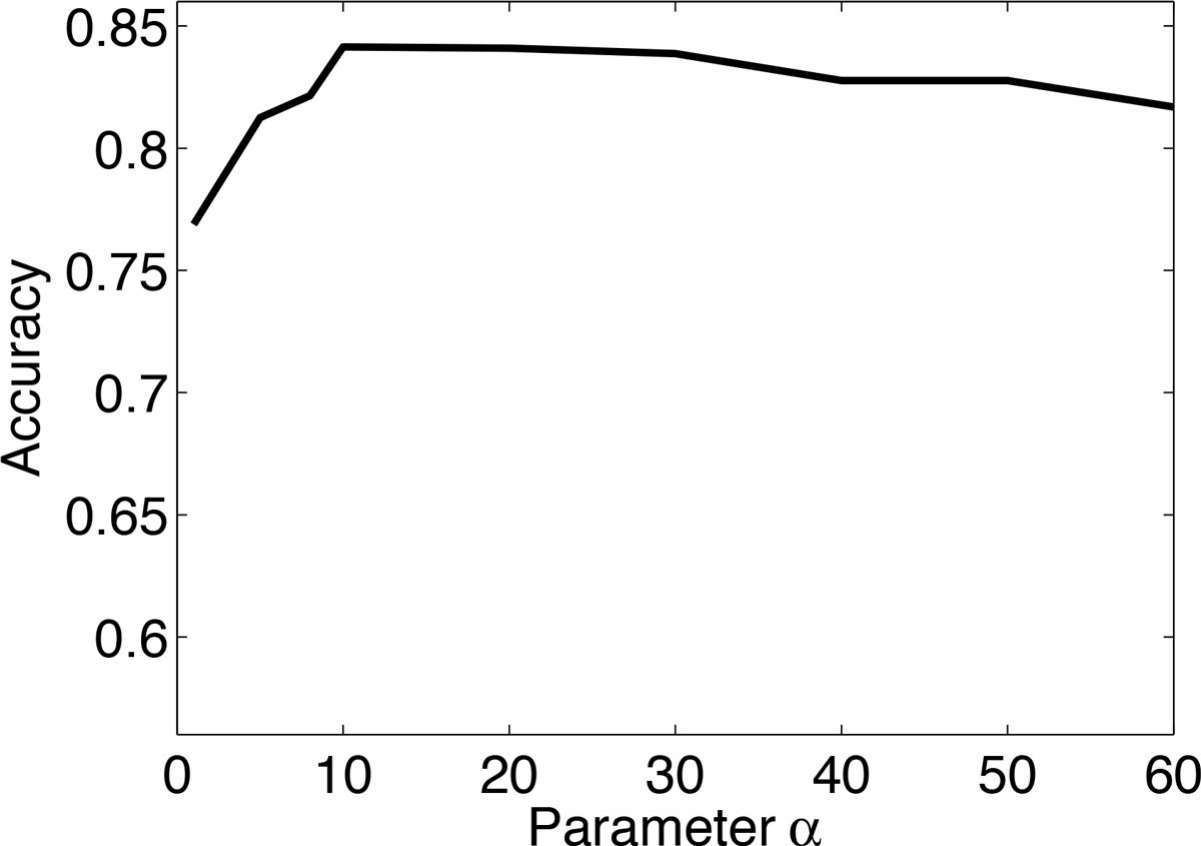
**Classification accuracy of RNMF with respect to different *α *for the problem (1) of KDD Cup 2001 dataset**. (the parameter *β *is fixed at 5).

**Figure 5:**
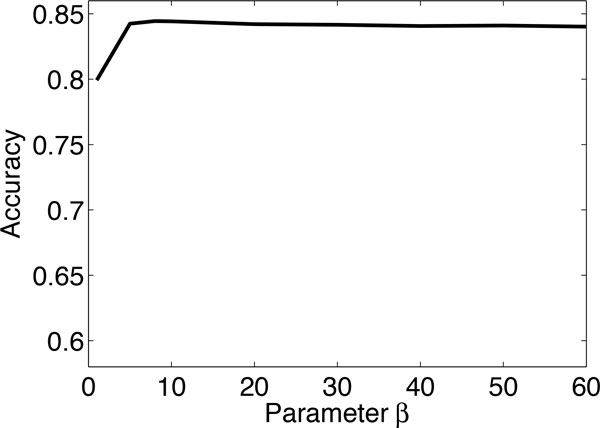
**Classification accuracy of RNMF with respect to different *β *for the problem (1) of KDD Cup 2001 dataset**. (the parameter *α *is fixed at 10).

In the proposed RNMF method, we need to set the the parameters *α *and *β *which quantify the importance of the label matrix factorization term and the network regularization term of the objective function in Eq. (11). In this experiment, we investigate how different values of the parameters *α *and *β *affect the classification accuracy of the proposed method. We examine the sensitivity of RNMF with respect to different *α *and *β*. (i) We fix *β *= 5 and vary *α*. Figure 4 shows the classification accuracy of RNMF against different values of *α *on problem (1) of the KDD Cup 2001 dataset. From the figure, we observe that when *α *is small the accuracy is poor because the RNMF algorithm boils down to an unsupervised NMF approach in this situation. The accuracy of the proposed RNMF method increases as the value of *α *increases, and the accurate for *α *between 5 to 60 does not change significantly. (ii) We fix *α *= 10 and vary *β*. Figure 5 shows the classification accuracy of RNMF against different values of *β*. One observes that when *β *is small, the classification accuracy is degraded, because no smoothness is used in this situation. As the parameter *β *increases, the accuracy reaches a plateau between 5 to 60, and does not change significantly. In summary, the experimental results show that one can use the method in a robust way across a wide range of parameters. The best performance is achieved at *α *= 10 and *β *= 5. Therefore, we set *α *= 10 and *β *= 5 as default values in the experiments.

### Interaction relations

The coefficient matrix **V **learnt by the proposed RNMF method can be used to estimate the interaction effects among the proteins. Given two protein instances *xi *and *x_j _*, their interaction can be estimated by the cosine similarity between their coefficient vectors **v***_i _*= [*v*_*i*,1_,...,*v*_*i*,*K*_] and **v***_j _*= [*v*_*j*,1_, ..., *v*_*j*,*K*_]. The resulting similarity ranges from 0 to 1, with 0 indicating the instances are independence, and 1 indicating the instances are highly interrelated. We apply the cosine similarity measure to evaluate the interaction relations of 5 randomly selected genes (G238510, G238510, G234935, G235158, G237021, G234980) to their interrelated genes in the KDD Cup 2001 dataset. Table 2 shows these interrelated proteins (discovered by previous studies) and their similarity values (computed by using the matrix **V**). In general, we can see that these interrelated genes tend to have large similarity values. This provides evidence of the advantages of using our proposed method to detect the interactions.

**Table 2:** Selected interrelated genes and their similarity computed by the proposed method.

GeneID	GeneID	Similarity
G238510	G239467	0.9984
G238510	G239178	0.9987
G238510	G235250	0.9983
G234935	G234445	0.9094
G234935	G239966	0.9388
G234935	G235763	0.9589
G234935	G235329	0.9700
G235158	G234735	0.9776
G235158	G234074	0.9808
G235158	G234177	0.9837
G235158	G235216	0.9554
G237021	G234486	0.8831
G237021	G234065	0.9222
G237021	G239804	0.9285
G237021	G239266	0.8751
G234980	G235439	0.9865
G234980	G235231	0.9843
G234980	G234914	0.9939
G234980	G235780	0.9305

### Results on protein function prediction

We also conduct experiments for problem (2) of KDD Cup 2001, i.e., the multi-label protein function prediction problem. We compare the proposed RNMF algorithms with baseline classifiers: SVM, wvRN+RN, ICA, semi-ICA and ICML. For SVM, wvRN+RN, ICA and semi-ICA, we use the binary relevance (BR) method [33] to decompose the multi-label problem into a set of *q *binary classification problems using one-against-all strategy, and train independent classifier for each single-label problem. The predictions for all *q *binary classification problems are combined to make the final prediction.

**Figure 6:**
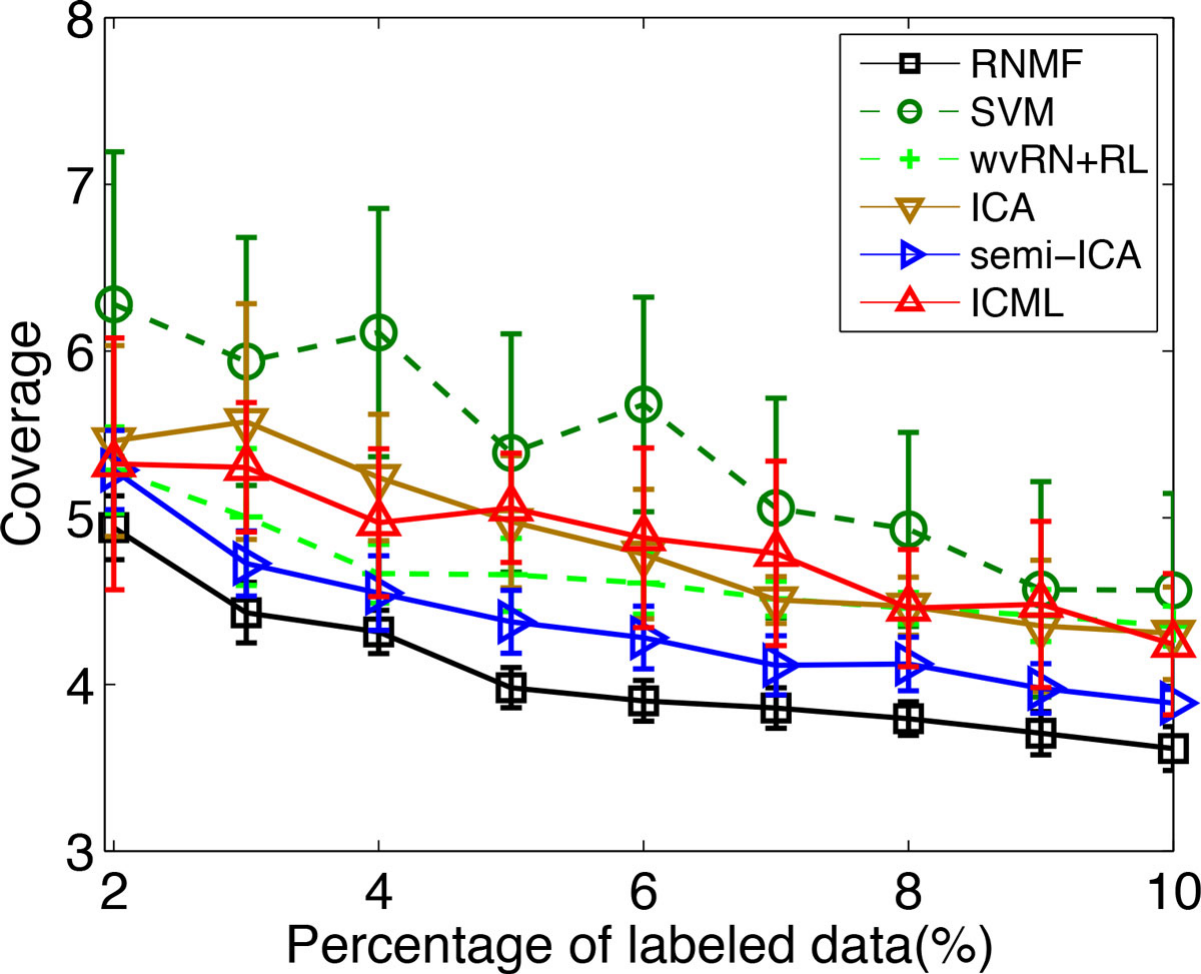
**Coverage of different algorithms with varying percentages of labeled data on problem (2) of KDD Cup 2001**.

**Figure 7:**
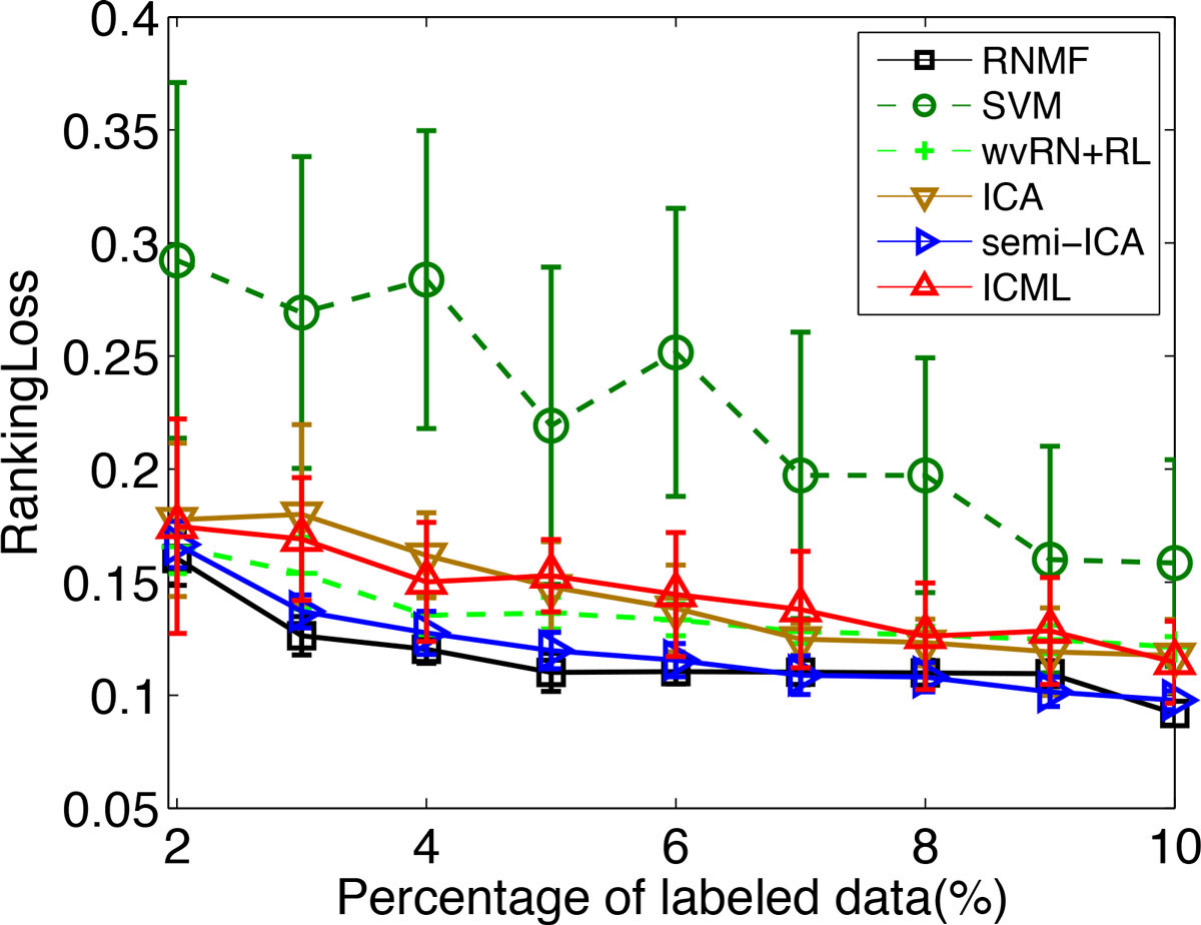
**RankingLoss of different algorithms with varying percentages of labeled data on problem (2) of KDD Cup 2001**.

We compare the performance of our proposed RNMF approach and other tested algorithms with varying percentages of labeled data from 2% to 10%. For each percentage, we execute each algorithm 10 times and report the results of mean as well as standard deviation of each compared algorithms over 10 runs. The results are shown in Figure 6 and 7 in terms of *Coverage *and *RankingLoss*, respectively. For these two evaluation metrics, the smaller the value of the metrics, the better the performance of the algorithms. From the experimental results, we see that the RNMF method (the black line) has the best performance (lies under the other curves) across different percentages of labeled data from 2% to 10%. This provides evidence of the advantage of the proposed RNMF method for multi-label protein function prediction.

## Conclusion

In this paper, we utilize a latent graph approach for finding an integration latent graph by exploiting various latent linkages and judiciously integrate the linkages to generate a latent graph to effectively propagate the label information from labeled data to unlabeled data. For protein function prediction, we developed a novel method, called regularized non-negative matrix factorization (RNMF), to seek a matrix factorization which respect the attribute features, latent graph, and unlabeled data for classification prediction. In RNMF, a label matrix factorization term and a network regularization term are incorporated into the NMF objective function to encode the network structure and label information. As such, the learnt RNMF has more discriminating power than the other compared baseline methods.

Several questions remain to be investigated in our future work: 1) A challenge problem is the lack of large benchmark datasets for evaluation the scalability of the proposed method. Future work includes collecting and generating large datasets for more extensive empirical study. 2) Using suitable parameters are critical to the RNMF model and the compared methods. We will further investigate how to select the parameters effectively and efficiently with limited number of labeled data. 3) The convergence proofs of the RNMF can follow the idea in the proofs of Lee and Seung's paper [17] for the original NMF. It is interesting to apply Lee and Seung's idea [17] to theoretically proof the convergence of the RNMF model. 4) Advances in biotechnology have generated a wide variety of heterogeneous biology networks. This suggests investigating the performance of the RNMF model on different networks.

## Competing interests

The authors declare that they have no competing interests.

## Authors' contributions

Q. Wu participated in designing the algorithm, conducting the experiments, and drafting the manuscript. Z. Wang, C. Li, Y. Ye, Y. Li and N. Sun revised and finalized the paper. All authors read and approved the final manuscript.

## Declarations

Publication of this article was funded by the corresponding author.

This article has been published as part of *BMC Systems Biology *Volume 9 Supplement 1, 2015: Selected articles from the Thirteenth Asia Pacific Bioinformatics Conference (APBC 2015): Systems Biology. The full contents of the supplement are available online at http://www.biomedcentral.com/bmcsystbiol/supplements/9/S1
